# Chemical coding of piglets small intestine neurons after prenatal exposure to β-hydroxy-β-methylbutyrate

**DOI:** 10.2478/jvetres-2025-0024

**Published:** 2025-04-19

**Authors:** Aleksandra Dajnowska, Katarzyna Kras, Ewa Tomaszewska, Piotr Dobrowolski, Renata Klebaniuk, Siemowit Muszyński, Marcin Bartłomiej Arciszewski

**Affiliations:** 1 Department of Animal Anatomy and Histology, Department of Animal Physiology, Institute of Animal Nutrition and Bromatology, Faculty of Veterinary Medicine, Department of Biophysics, Faculty of Environmental Biology, University of Life Sciences in Lublin, 20-950 Lublin, Poland; 2 Department of Animal Physiology, Institute of Animal Nutrition and Bromatology, Faculty of Veterinary Medicine, Department of Biophysics, Faculty of Environmental Biology, University of Life Sciences in Lublin, 20-950 Lublin, Poland; 3 Department of Functional Anatomy and Cytobiology, Maria Curie-Skłodowska University, 20-033 Lublin, Poland; 4 Institute of Animal Nutrition and Bromatology, Faculty of Veterinary Medicine, Department of Biophysics, Faculty of Environmental Biology, University of Life Sciences in Lublin, 20-950 Lublin, Poland; 5 Department of Biophysics, Faculty of Environmental Biology, University of Life Sciences in Lublin, 20-950 Lublin, Poland

**Keywords:** ENS, HMB, pigs, prenatal supplementation, small intestine

## Abstract

**Introduction:**

The global swine industry faces significant challenges related to improving the survival and health of newborn piglets. Attention has come to β-hydroxy-β-methylbutyrate (HMB), a metabolite of leucine, for its potential in prenatal nutritional programming in sows, which can improve piglet body weight and support the development of the skeletal and digestive systems. The effects of prenatal HMB supplementation were investigated on the chemical coding of the enteric nervous system (ENS) in the small intestine of neonatal piglets.

**Material and Methods:**

The experiment was conducted on piglets from 12 sows divided into a control and an experimental group. Sows in the experimental group received HMB at a dose of 0.2 g/kg body weight per day from day 70 to 90 of gestation. After parturition, one piglet from each litter was euthanised and parts of the duodenum, jejunum and ileum were exsected. Tissue sections were fixed in paraffin, reacted with anti–vasoactive intestinal peptide (VIP), anti–cocaine- and-amphetamine-regulated transcript (CART), anti–neuronal nitric oxide synthase (nNOS) and anti-substance P (SP) antibodies, and the immunoexpression of VIP, CART, nNOS and SP was determined histomorphometrically by calculating the area of fibres which were immunoreactive with each.

**Results:**

Supplementation with HMB in sows caused significant changes in the ENS of newborn piglets, including an increase in the area of fibres reactive to CART and nNOS in certain layers and sections of the small intestine, and a decrease in the area of fibres reactive to SP and VIP.

**Conclusion:**

The results indicate that prenatal supplementation with HMB in sows may significantly influence the functioning of the gastrointestinal tract in newborn piglets.

## Introduction

To achieve high pork production efficiency and generate significant economic profits, the global swine industry must confront numerous challenges. These include the need to optimise nutrition, implement effective disease control and prevention measures, maintain proper hygiene and safety, and address issues such as extreme temperature fluctuations and low survival rates among newborn piglets (8, 24). The latter issue is closely linked to the optimisation of nutrition, which, beyond its obvious benefits in pig production, can also help address piglets’ general health status (18). The optimal nutrition of pregnant sows with a diet that is properly balanced and rich in key nutrients significantly improves reproductive performance, birth litter weight, weaning weight and the survival rate of newborn piglets. The composition of colostrum and milk is regulated by the mother’s diet, which directly influences the maturation of the newborns’ immune systems, providing protection against infections and supporting the development of a healthy gut microbiome. Overall intestinal health is, in turn, extremely important in combating piglet weaning stress, which is often combined with diarrhoea (11). However, the shaping of the offspring’s health starts earlier, in prenatal life. The prenatal programming theory states that postnatal development is shaped not only by genetics but also by prenatal non-genetic factors, such as the nutrients present in the pregnant female’s diet (2). One supplement that has been successfully used in prenatal nutritional programming is β-hydroxy-β-methylbutyrate (HMB) (26). This metabolite of leucine is commonly used as a dietary supplement to support protein metabolism, insulin activity and skeletal muscle hypertrophy. It is particularly popular among athletes and physically active individuals. In animals, HMB supplementation has shown various benefits, particularly in livestock, where it primarily leads to increased weight gain and improved production yields (29). Studies on pigs have demonstrated that HMB supplementation in sows’ diets as part of nutritional programming leads to heavier newborn piglets and faster attainment of market weight, because more protein is synthesised in the piglets’ skeletal muscles. Positive effects on the development of the offspring’s skeletal and digestive systems have also been observed (2, 5, 27). Additionally, it was noted that HMB may improve the uniformity of piglet birth weights, potentially mitigating the effects of intrauterine growth restriction (3).

As noted earlier, gut health plays a vital role in piglet development. The enteric nervous system (ENS), a key part of the digestive tract, is crucial for the early development of digestive functions. Intestinal innervation starts prenatally and continues to mature after birth, which is essential for newborn piglets as they transition to solid food (13). It is already known that HMB supplementation in pregnant sows significantly affects the expression of leptin, vasoactive intestinal peptide (VIP), and other gut barrier proteins in the offspring during weaning, while having a lesser impact on the basic morphology of the small intestine (26). There is presently insufficient data on the effect of HMB on the prenatal development of small intestine innervation in newborn pigs. Consequently, it is not known whether the effects observed by Tomaszewska *et al*. (26) were present from birth. Based on the available research, we hypothesise that HMB supplementation in pregnant sows will affect the chemical coding of the ENS in their newborn offspring. To verify this hypothesis, this study analyses the VIP, cocaine-and-amphetamine-regulated transcript (CART), neuronal nitric oxide synthase (nNOS) and substance P (SP) peptides in the small intestine of neonatal piglets after prenatal HMB supplementation, and evaluates the surface area of nerve fibres reacting with these proteins in comparison to piglets without prenatal supplementation.

## Material and Methods

### Animal welfare

Throughout the entire experiment, the health of both pregnant sows and newborn piglets was closely monitored by a veterinarian. Regular veterinary care ensured the welfare of the animals and eliminated any undesirable factors that could affect the course of the experiment. Additionally, this study complied with the requirements of Directive 2010/63/EU of the European Parliament and of the Council on the protection of animals used for scientific purposes, and was approved by the Local Ethics Committee for Animal Experiments at the University of Life Sciences in Lublin (protocol No. 2014/29).

### Animals, breeding and experimental design

The study involved 24 piglets born from 12 healthy primiparous sows of the Large White Polish breed, approximately 10 months old. A dose of 0.2 g/kg of body weight (b.w.) of HMB (Lonza, Basel, Switzerland) was administered with the morning meal to six randomly selected sows daily from the 70^th^ to 90^th^ day of gestation. The supplementation period and HMB dose were selected based on previous studies (23, 25). The remaining six sows were fed a standard diet. Both groups of sows were housed in standard conditions with controlled diets, including balanced commercial feed for pregnant and lactating sows, provided in the amount of 3.0 kg of feed mixture per sow, with water given *ad libitum* (7). One kilogram of feed mixture contained 13.2 megajoules of metabolisable energy, 162 g of crude protein, 9.5 g of lysine, and essential amino acids with provision of 100% of lysine requirement, 60% of methionine + cysteine requirement, 65% of threonine requirement and 225% of tryptophan requirement. The gestation length was the same between the control and experimental groups, and the piglets were born *via* physiological delivery without complications. No congenital defects or stillbirths were observed among the offspring. Randomly selected piglets were euthanised *via* intravenous injection of lethal doses of pentobarbitalum natricum (Morbital; Biowet, Puławy, Poland). For this purpose, 12 piglets (6 male and 6 female) were taken from each group.

### Tissue collection and immunohistochemical analysis

Ten-millimetre fragments of the small intestine (the duodenum, jejunum and ileum) were collected from each piglet. The tissues were fixed and immunohistochemical reactions were conducted according to a previously described protocol (4). The antibodies used in the study are described in Table 1.

**Table 1.** Primary and secondary antibodies used in the study

Antibody	Host	Catalogue number	Dilution	Manufacturer
Primary antibody				
anti-VIP	rabbit	ab22736	1:400	Abcam Limited, Cambridge, UK
anti-CART	rabbit	H-003-62	1:2,000	Phoenix Pharmaceuticals, Burlingame, CA, USA
anti-nNOS	rabbit	160870	1:200	Cayman Chemical, Ann Arbor, MI, USA
anti-SP	mouse	ab14184	1:1,000	Abcam Limited, Cambridge, UK
Secondary antibody				
anti-mouse/anti-rabbit	goat	DPVB-HRP	RTU	ImmunoLogic, Duiven, the Netherlands

1 VIP – vasoactive intestinal peptide; CART – cocaine-and-amphetamine-regulated transcript; nNOS – neuronal nitric oxide synthase; SP – substance P;

1 HRP – horseradish peroxidase-conjugated; RTU – ready to use

### Histomorphometry analysis

The sections were examined under a light microscope (BX-51 DSU; Olympus, Tokyo, Japan) equipped with a digital camera (DP-70; Olympus) at magnifications of 10×, 20× and 40×. No positive immunoreactivity was observed on any of the slides subjected to the negative control reaction. High-resolution digital images were taken, all by a single person, using Cell^M 2.3 in cellSens Standard software (Olympus) under consistent lighting conditions and uniform brightness and contrast settings. Surface area analysis of nerve fibres was performed using ImageJ version 1.54f software (20), including measurements of the area of CART-, nNOS-, SP- and VIP-immunoreactive nerve fibres across the entire cross-section of the duodenum, jejunum and ileum from each piglet in the experimental group (n = 12) and in the control group (n = 12). The area of nerve fibres immunoreactive to nNOS, SP, CART and VIP was expressed as the ratio of the area occupied by the fibres in μm^2^ to 100 μm^2^ of the intestinal cross-section.

### Statistical analysis

To assess the effect of HMB supplementation on the chemical coding of the small intestine ENS in piglets, a statistical analysis of the obtained results was performed. Student’s *t*-test was used to compare the mean surface areas of CART-, nNOS-, SP- and VIP-immunoreactive nerve fibres between the experimental group (HMB) and the control group. This test allowed for the evaluation of the statistical significance of differences between the groups. The analyses were conducted using GraphPad Prism software, version 9.5.1 for Windows (GraphPad Software, San Diego, CA, USA). A P-value < 0.05 was considered statistically significant.

## Results

### Muscular layer of the duodenum

A statistically significant increase in the area of CART-reactive fibres in this tissue was observed in the group of piglets supplemented with HMB compared to the control group (P-value < 0.001). However, a significant decrease in the area was noted for SP- and VIP-reactive fibres in the experimental group, with P-values of 0.002 and <0.001, respectively. No significant difference was found in the area of nNOS-reactive fibres (P-value = 0.240) between the groups.

### Submucosa of the duodenum

In the submucosa of the duodenum, a significantly smaller area of VIP-reactive fibres (P-value < 0.001) was noted in the experimental group of piglets, with no significant changes in the area of CART-, SP- or nNOS-reactive fibres (P-values of 0.201, 0.732, and 0.100, respectively) compared to the control group.

### Mucosa of the duodenum

A significant contraction of the area of CART-, SP- and VIP-reactive fibres was seen in the experimental group of piglets (P-value < 0.001 each). Additionally, a significant expansion of the area of nNOS-reactive fibres (P-value < 0.001) was observed in the same group compared to the control group.

### Muscular layer of the jejunum

This tissue presented a statistically significantly lesser area of SP-, VIP- and nNOS-reactive fibres in the experimental group of piglets, with P-values of 0.002, <0.001 and 0.039, respectively. However, no significant difference was found in the area of CART-reactive fibres (P-value = 0.187) compared to the control group.

### Submucosa of the jejunum

In the submucosa of the jejunum, the area of VIP- and nNOS-reactive fibres (P-value < 0.001 each) was observed to be significantly smaller in the experimental group of piglets, while no significant differences were found between the areas of CART- or SP-reactive fibres (P-values of 0.337 and 0.224, respectively) and those of the control group.

### Mucosa of the jejunum

In these sections, the CART-(P-value < 0.001), SP-(P-value = 0.001) and VIP-reactive fibres (P-value = 0.002) extended over less area in the experimental group of piglets, while the area of nNOS-reactive fibres occupied more tissue than in the control group (P-value < 0.001).

### Muscular layer of the ileum

The muscular layer of the ileum revealed a significant increase in the area of CART-reactive fibres (P-value < 0.001) in the experimental group of piglets, along with a significant decrease in the area of SP- and VIP-reactive fibres (P-values of 0.017 and <0.001, respectively). No significant difference was found in the area of nNOS-reactive fibres (P-value = 0.280) between the experimental and control groups.

### Submucosa of the ileum

The experimental group of piglets’ submucosa of the ileum presented a significantly larger area of CART-reactive fibres (P-value = 0.015) and a significantly smaller one of VIP-reactive fibres (P-value = 0.004), but displayed areas of SP- and nNOS-reactive fibres not significantly different (P-values of 0.128 and 0.113, respectively) from those of the control group.

**Fig. 1.**
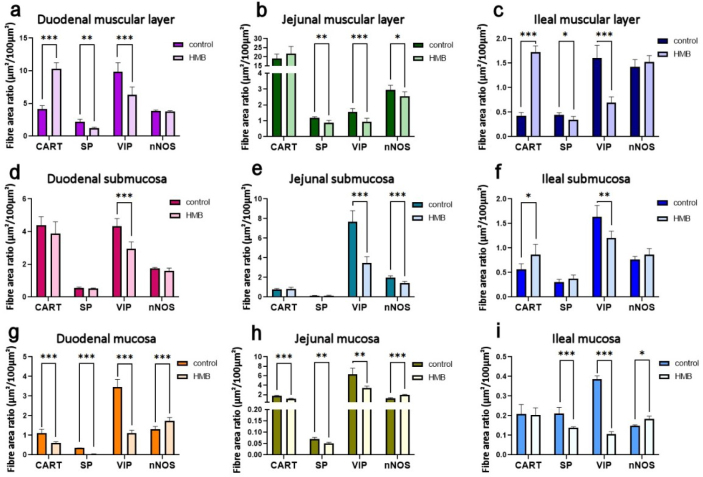
Prenatal effect of β-hydroxy-β-methylbutyrate (HMB) supplementation on the area occupied by cocaine-and-amphetamine-regulated transcript (CART)-immunoreactive (IR), substance P (SP)-IR, vasoactive intestinal peptide (VIP)-IR, and neuronal nitric oxide synthase (nNOS)-IR nerve fibres in the muscular layer of the duodenum, jejunum and ileum (a, b and c), the submucosa of the duodenum, jejunum and ileum (d, e and f), and the mucosa of the duodenum, jejunum and ileum (g, h and i) in newborn piglets prenatally supplemented with HMB and control piglets. * – significant difference between the control and HMB groups at P-value < 0.05; ** – at P-value < 0.01; *** – at P-value < 0.001

**Fig. 2.**
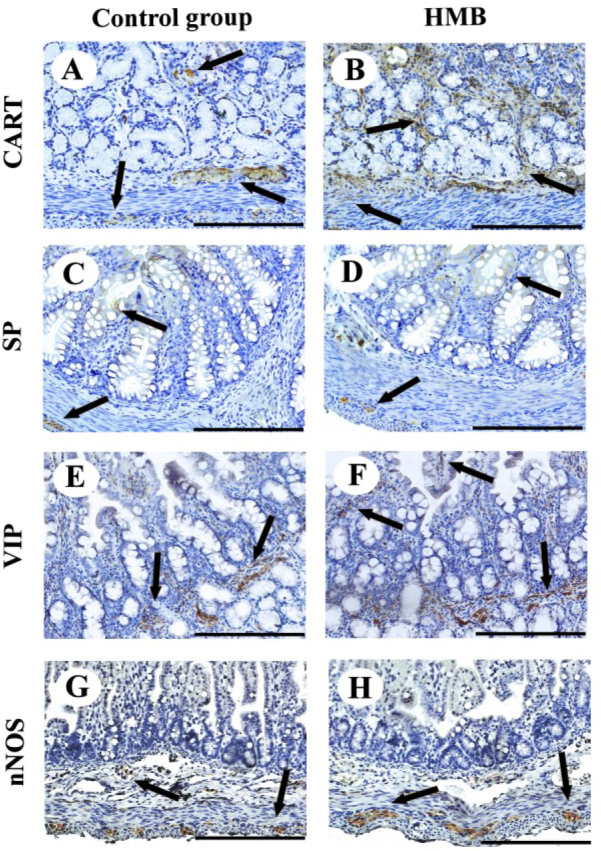
Representative photomicrographs of the immunohistochemical reactions for cocaine-and-amphetamine-regulated transcript (CART) (A and B), substance P (SP) (C and D), vasoactive intestinal peptide (VIP) (E and F) and neuronal nitric oxide synthase (nNOS) (G and H) in the duodenum (A, B, E and F) and jejunum (C, D, G and H) of control (A, C, E and G) and prenatally HMB-exposed (B, D, F and H) piglets. Arrow – positive reaction. Scale bar = 200 μm

### Mucosa of the ileum

Sections of the mucosa of the ileum were noted to have a significant decrease in the area of SP-(P-value < 0.001) and VIP-reactive fibres (P-value < 0.001) and a significant increase in the area of nNOS-reactive fibres (P-value = 0.002) in the experimental group of piglets compared to the control group.

## Discussion

The present study investigated the effects of prenatal HMB supplementation on the chemical coding of the ENS in the small intestine of neonatal piglets, focusing on the immunoexpression of VIP, CART, nNOS and SP peptides. There is limited information regarding how HMB influences the prenatal development of intestinal innervation, and whether the changes observed in previous studies manifest from birth. The results of the conducted study confirmed the initial assumption that prenatal HMB supplementation would affect the chemical coding of the ENS.

One of the most consistent findings across intestinal regions and layers was the significant decrease in VIP- and SP-reactive fibres in piglets prenatally supplemented with HMB, especially in the muscular layers and mucosa. These neuropeptides play critical roles in the regulation of intestinal motility, secretion and inflammation (15). Vasoactive intestinal peptide acts neuroprotectively and to counter inflammation, reducing the expression of pro-inflammatory cytokines and enlarging the production of anti-inflammatory cytokines (21). It also participates in the modulation of intestinal motility and blood flow (19), which affects digestion and protects the intestines from inflammatory conditions (6). Substance P, another neuropeptide, is in the tachykinin family and acts as a neurotransmitter and neuromodulator in the gastrointestinal tract (12). It modulates immune, vascular and motor responses and serves as a pro-inflammatory mediator by activating immune cells like mast cells, macrophages and T cells *via* the NK1 receptor (17). This activation leads to the release of cytokines and chemokines triggering inflammation and diarrhoea and stimulating intestinal motility. Production of SP is stimulated by IL-12 and inhibited by IL-10 and transforming growth factor β. Levels of SP rise with inflammation in inflammatory bowel disease, being higher in ulcerative colitis compared to Crohn’s disease and indicating a link between elevated SP levels and greater inflammation (17). The reduction in VIP- and SP-IR fibres may suggest that prenatal HMB supplementation possibly inhibits the activity of these pathways, potentially affecting gastrointestinal motility and immune responses in newborns. However, it is important to consider that the ENS in newborn pigs is still in a developmental phase, which means that changes in VIP and SP fibre density may not necessarily indicate impairment of the ENS due to HMB supplementation (8). Given that the ENS is undergoing maturation during this period (13), a decrease in VIP and SP may suggest a shift in the regulatory mechanisms that control intestinal function (28), possibly facilitating adaptation to postnatal life. Therefore, while the observed decrease in VIP and SP fibres raises questions about the influence of HMB on gut physiology, it does not automatically imply that HMB supplementation negatively impacts the development or functionality of the ENS in newborn pigs. Unknown mechanisms specific to newborns may be in play here, which require further investigation.

There is a similar study that seemingly contradicts our results. Tomaszewska *et al*. (26) demonstrated an increased level of VIP-IR fibres in the submucosal plexus of weaned piglets prenatally supplemented with HMB. The course of the study was the same as ours; however, the difference was the age of the animals, where weaned piglets were enrolled by Tomaszewska *et al*. (26) and newborns by us. Such a result is most likely a reflection of the differing level of development in the ENS of the newborns in our study compared to the older animals. These contrasting outcomes reinforce the concept that the ENS undergoes dynamic, stage-specific shifts in neurochemical coding, which shape how prenatal HMB supplementation impacts gut physiology over time.

In comparison to SP- and VIP-reactive fibres, CART-reactive fibres extended over significantly more of the duodenal and ileal muscular layer and ileal submucosa areas. Cocaine-and-amphetamine-regulated transcript, known to be involved in regulating appetite and energy homeostasis, has been less studied in the context of the ENS (30). The proliferation of CART-reactive fibres suggests that HMB might influence the energy regulatory mechanisms of the gut (16), possibly improving nutrient absorption or promoting energy efficiency in neonatal piglets. Fibres reactive with nNOS also showed interesting patterns, particularly in the mucosa, where there was a consistent increase across all intestinal regions. Neuronal NOS is a marker for nitrergic neurons, which are essential for smooth muscle relaxation and regulation of intestinal motility *via* the production of NO (14). The observed increase in nNOS-reactive fibres in the mucosa, coupled with a lack of significant changes in the muscular layers (except in the jejunum), suggests that prenatal HMB supplementation might enhance nitrergic signalling in the mucosa, possibly improving mucosal blood flow and barrier function (14). Nitric oxide has well-known vasodilatory effects, which could enhance nutrient absorption and immune defence mechanisms in the intestinal mucosa (14).

Studies describing the impact of the toxin fumonisin and the toxic substance bisphenol A on the expression of the neuropeptides we investigated showed results opposite to ours, namely an increase in VIP- and SP-positive fibres and a decrease in CART- and nNOS- positive fibres (10, 23). In contrast, HMB as a supplement appears to regulate the expression of these neurotransmitters, which may indicate its protective effects. The decrease in SP and VIP levels following HMB administration could suggest a reduction in inflammation and an improvement in intestinal function, contrasting with the effect of toxins and toxic substances, which potentiates the activity of these peptides in response to the resultant damage (10, 22). The response to HMB supplementation varied across different regions of the small intestine. In the duodenum, a significant accrual of CART-reactive fibres in the muscular layer and a significant reduction in VIP-reactive fibres in both the submucosa and mucosa indicate a strong modulatory effect of HMB on the upper intestinal segments. In the ileum, however, HMB supplementation resulted in a marked increase in both CART-reactive fibres and nNOS-reactive fibres in the mucosa, suggesting that the effects of HMB may intensify towards the distal small intestine.

These regional differences could reflect the varying functional roles of the small intestine. The duodenum is primarily responsible for the initial stages of digestion and absorption, while the ileum plays a key role in the absorption of bile acids, vitamin B12 and other nutrients (9). The increased presence of CART and nNOS in the ileum might suggest a role for HMB in enhancing nutrient absorption and modulating motility towards the distal regions of the gut, where nutrient content is lower and energy absorption may be more critical. These findings provide new insights into how prenatal HMB supplementation may shape the development of the ENS and, by extension, gastrointestinal function in neonatal piglets. The reduction in SP- and VIP-reactive fibres, alongside the accrual of CART- and nNOS-reactive fibres, suggests that HMB exerts a selective influence on neurochemical pathways involved in gut motility, energy regulation and possibly immune function.

## Conclusion

The beneficial effects of HMB on muscle preservation, immune modulation and anti-inflammatory responses in other tissues are known (1). The observed changes may reflect similar protective effects, potentially safeguarding the developing gut from inflammatory or oxidative damage during critical stages of growth. This could be particularly important for neonatal animals, as early-life gastrointestinal development has long-term consequences for health and productivity (31). However, the mechanisms by which HMB influences ENS development remain speculative. Further research is needed to elucidate the molecular pathways involved, potentially including interactions with growth factors, inflammatory mediators and oxidative stress responses, which are known to play a role in gut development and health.

## References

[1] Arazi H., Taati B., Suzuki K. (2018). A Review of the Effects of Leucine Metabolite (β-Hydroxy-β-Methylbutyrate) Supplementation and Resistance Training on Inflammatory Markers: A New Approach to Oxidative Stress and Cardiovascular Risk Factors. Antioxidants.

[2] Blicharski T., Tomaszewska E., Dobrowolski P., Hułas-Stasiak M., Muszyński S. (2017). A metabolite of leucine (β-hydroxy-β-methylbutyrate) given to sows during pregnancy alters bone development of their newborn offspring by hormonal modulation. PLoS One.

[3] Clarke A.S., Faulk C., Shurson G.C., Gallaher D.D., Johnston L.J. (2023). Evaluation of Feeding Beta-Hydroxy-Beta-Methylbutyrate (HMB) to Mouse Dams during Gestation on Birth Weight and Growth Variation of Offspring. Animals.

[4] Dajnowska A., Osiak-Wicha C., Piech M., Muszyński S., Tomaszewska E., Ropka-Molik K., Krzysiak M.K., Arciszewski M.B. (2023). Immunoexpression of Spexin in Selected Segments of the Bovine (*Bos taurus taurus*) Gastrointestinal Tract. Animals.

[5] Dobrowolski P., Muszyński S., Donaldson J., Jakubczak A., Żmuda A., Taszkun I., Rycerz K., Mielnik-Błaszczak M., Kuc D., Tomaszewska E. (2021). The Effects of Prenatal Supplementation with β-Hydroxy-β-Methylbutyrate and/or Alpha-Ketoglutaric Acid on the Development and Maturation of Mink Intestines Are Dependent on the Number of Pregnancies and the Sex of the Offspring. Animals?.

[6] Eklund S., Jodal M., Lundgren O., Sjöqvist A. (1979). Effects of vasoactive intestinal polypeptide on blood flow, motility and fluid transport in the gastrointestinal tract of the cat. Acta Physiol Scand.

[7] Grela E.R., Skomiał J. (2015). Zalecenia żywieniowe i wartość pokarmowa pasz dla świń, II wydanie (Nutritional recommendations and nutritional value of feed for pigs, Second Edition – in Polish).

[8] Ji Y., Wu Z., Dai Z., Wang X., Li J., Wang B., Wu G. (2017). Fetal and neonatal programming of postnatal growth and feed efficiency in swine. J Anim Sci Biotechnol.

[9] Kiela P.R., Ghishan F.K. (2016). Physiology of Intestinal Absorption and Secretion. Best Pract Res Clin Gastroenterol.

[10] Kras K., Rudyk H., Muszyński S., Tomaszewska E., Dobrowolski P., Kushnir V., Muzyka V., Brezvyn O., Arciszewski M.B., Kotsyumbas I. (2022). Morphology and Chemical Coding of Rat Duodenal Enteric Neurons Following Prenatal Exposure to Fumonisins. Animals.

[11] Li Q., Yang S., Zhang X., Liu X., Wu Z., Qi Y., Guan W., Ren M., Zhang S. (2021). Maternal Nutrition During Late Gestation and Lactation: Association With Immunity and the Inflammatory Response in the Offspring. Front Immunol.

[12] Mazzoni M., Cabanillas L., Costanzini A., Caremoli F., Million M., Larauche M., Clavenzani P., Giorgio R.D., Sternini C. (2023). Distribution, quantification, and characterization of substance P enteric neurons in the submucosal and myenteric plexuses of the porcine colon. Cell and Tissue Res.

[13] Modina S.C., Aidos L., Rossi R., Pocar P., Corino C., Di Giancamillo A. (2021). Stages of Gut Development as a Useful Tool to Prevent Gut Alterations in Piglets. Animals.

[14] Mu K., Yu S., Kitts D.D. (2019). The Role of Nitric Oxide in Regulating Intestinal Redox Status and Intestinal Epithelial Cell Functionality. Int J Mol Sci.

[15] O’Connor T.M., O’Connell J., O’Brien D.I., Goode T., Bredin C.P., Shanahan F. (2004). The Role of Substance P in Inflammatory Disease. J Cell Physiol.

[16] Oponowicz A., Kozłowska A., Gonkowski S., Godlewski J., Majewski M. (2018). Changes in the Distribution of Cocaine-and Amphetamine-Regulated Transcript-Containing Neural Structures in the Human Colon Affected by the Neoplastic Process. Int J Mol Sci.

[17] Patel M., Subas S.V., Ghani M.R., Busa V., Dardeir A., Marudhai S., Cancarevic I. (2020). Role of Substance P in the Pathophysiology of Inflammatory Bowel Disease and Its Correlation With the Degree of Inflammation. Cureus.

[18] Rodrigues L.A., Koo B., Nyachoti M., Columbus D.A. (2022). Formulating Diets for Improved Health Status of Pigs: Current Knowledge and Perspectives. Animals.

[19] Rząp D., Czajkowska M., Całka J. (2020). Neurochemical Plasticity of nNOS-, VIP-and CART-Immunoreactive Neurons Following Prolonged Acetylsalicylic Acid Supplementation in the Porcine Jejunum. Int J Mol Sci.

[20] Schneider C.A., Rasband W.S., Eliceiri K.W. (2012). NIH Image to ImageJ: 25 years of image analysis. Nat Methods.

[21] Sigalet D.L., Wallace L.E., Holst J.J., Martin G.R., Kaji T., Tanaka H., Sharkey K.A. (2007). Enteric neural pathways mediate the anti-inflammatory actions of glucagon-like peptide 2. Am J Physiol Gastrointest Liver Physiol.

[22] Szymanska K., Gonkowski S. (2018). Bisphenol A-Induced changes in the enteric nervous system of the porcine duodenum. NeuroToxicology.

[23] Świetlicka I., Muszyński S., Tomaszewska E., Dobrowolski P., Kwaśniewska A., Świetlicki M., Skic A., Gołacki K. (2016). Prenatally administered HMB modifies the enamel surface roughness in spiny mice offspring: An atomic force microscopy study. Arch Oral Biol.

[24] Theil P.K., Lauridsen C., Quesnel H. (2014). Neonatal piglet survival: impact of sow nutrition around parturition on fetal glycogen deposition and production and composition of colostrum and transient milk. Animal.

[25] Tomaszewska E., Dobrowolski P., Świetlicka I., Muszyński S., Kostro K., Jakubczak A., Taszkun I., Żmuda A., Rycerz K., Blicharski T., Jaworska-Adamu J. (2018). Effects of maternal treatment with β-hydroxy-β-metylbutyrate and 2-oxoglutaric acid on femur development in offspring of minks of the standard dark brown type. J Anim Physiol Anim Nutr.

[26] Tomaszewska E., Prost Ł., Dobrowolski P., Chand D.K.P., Donaldson J., Czech A., Klebaniuk R., Fabjanowska J., Muszyński S. (2022). Prenatal programming of the small intestine in piglets: the effect of supplementation with 3-hydroxy-3-methylbutyric acid (HMB) in pregnant sows on the structure of jejunum of their offspring. Ann Anim Sci.

[27] Tomczyk-Warunek A., Blicharski T., Jarecki J., Dobrowolski P., Muszyński S., Tomaszewska E., Rovati L.C. (2021). The effect of maternal HMB supplementation on bone mechanical and geometrical properties, as well as histomorphometry and immunolocalization of VEGF, TIMP2, MMP13, BMP2 in the bone and cartilage tissue of the humerus of their newborn piglets. PLoS One.

[28] Tomita R. (2009). Regulation of Vasoactive Intestinal Peptide and Substance P in the Human Pyloric Sphincter. Hepato-gastroenterology.

[29] Wan H.F., Zhu J.T., Shen Y., Xiang X., Yin H.J., Fang Z.F., Che L.Q., Lin Y., Xu S.Y., Feng B., Wu D. (2016). Effects of Dietary Supplementation of β-Hydroxy-β-Methylbutyrate on Sow Performance and mRNA Expression of Myogenic Markers in Skeletal Muscle of Neonatal Piglets. Reprod Domest Anim.

[30] Zalecki M., Plywacz A., Antushevich H., Franke-Radowiecka A. (2021). Cocaine and Amphetamine Regulated Transcript (CART) Expression Changes in the Stomach Wall Affected by Experimentally Induced Gastric Ulcerations. Int J Mol Sci.

[31] Zhang Y., Choi S.H., Nogoy K.M., Liang S. (2021). Review: The development of the gastrointestinal tract microbiota and intervention in neonatal ruminants. Animal.

